# Temperature-Dependent Stiffening and Inelastic Behavior of Newly Synthesized Fiber-Reinforced Super Flexible Silica Aerogels

**DOI:** 10.3390/ma12182878

**Published:** 2019-09-06

**Authors:** Ameya Rege, Pascal Voepel, Emrah Okumus, Markus Hillgärtner, Mikhail Itskov, Barbara Milow

**Affiliations:** 1 Institute of Materials Research, German Aerospace Center, Linder Höhe, 51147 Cologne, Germany (E.O.) (B.M.); 2 Department of Continuum Mechanics, RWTH Aachen University, Kackertstr. 9, 52072 Aachen, Germany (M.H.) (M.I.)

**Keywords:** super flexible aerogel, fiber-reinforcement, insulation, temperature dependency, mechanical properties

## Abstract

In recent years, flexible silica aerogels have gained significant attention, owing to their excellent thermal and acoustic insulation properties accompanied by mechanical flexibility. Fiber reinforcement of such aerogels results in a further enhancement of the strength and durability of the composite, while retaining the excellent insulation properties. In this paper, the influence of four different kinds of fibers within a flexible silica aerogel matrix is studied and reported. First, a description of the synthesis procedure and the resulting morphology of the four aerogel composites is presented. Their mechanical behavior under uniaxial quasi-static tension and compression is investigated, particularly their performance under uniaxial compression at different temperature conditions (50 °C, 0 °C, and −50 °C). The reinforcement of the flexible silica aerogels with four different fiber types only marginally influences the thermal conductivity but strongly enhances their mechanical properties.

## 1. Introduction

Current aircraft cabin insulation is realized by means of packages of silica fibers with different grammages for both thermal and acoustical insulation. Facing a total temperature drop from the inside of the cabin to the outer skin of the aircraft of up to 80 °C, these insulation materials have to work very efficiently with minimum weight gain to the whole system. Although the current material exhibits good performances in terms of thermal insulation, several drawbacks can be observed. One of the main problems is the condensation of humidity within the insulation material and the subsequent ice formation. The formation of ice significantly drops the thermal insulation performance leading to higher energy (fuel) consumption for the cabin heating. Reduced turnaround times will lead to a more cost efficient flight plan. The effort, which is currently put into the reduction of turnaround times, was summarized by M. Schmidt [1]. However, shorter turnaround times will cause the lack of ice melting within the insulation material which then might lead to a phenomenon called “rain in the plane” [2]. In that case, the ice formed in the insulation material will melt during the flight and literally rain down on passengers. Besides these comfort-related problems, the ice formation within the insulation material also affects the economic and safety issues, as the additional weight increases the fuel consumption and the risk of corrosion [3].

Silica-based aerogels have gained significant interest given their outstanding thermal properties. The thermal conductivity of silica aerogels is approximately 40% to 70% lower than that of commonly used insulating materials, e.g., expanded polystyrene (EPS) (silica aerogel: 0.01–0.03 W m^−1^ K^−1^; EPS 0.035–0.05 W m^−1^ K^−1^) [4,5]. Although having been well investigated since their discovery in 1931 [6], silica aerogels are still in the focus of current research, since both fundamental understanding and material engineering are becoming more important. The potential of aerogels in aeronautical and astronautical applications has been previously outlined in the literature, either as thermal insulators or high velocity particle captors [7,8] or even in high-temperature energy storage applications [9]. The primary bottleneck in the application of silica aerogels in aeronautics has been their poor mechanical properties. New silica derivatives are being developed, as the properties of the final material can be tuned by the choice of precursors, which open novel fields of applications. For example, the working group of Nakanishi reported the synthesis and characterization of a new material which exhibits superflexibility, transparency and super insulating behavior [10]. Also machinability could be proven [11]. Another important topic in the current research is the fundamental understanding of the influence of process parameters. In particular, studies pertaining to heat treatment and ambient pressure drying increase the level of understanding [12,13,14].

**Figure 1:**
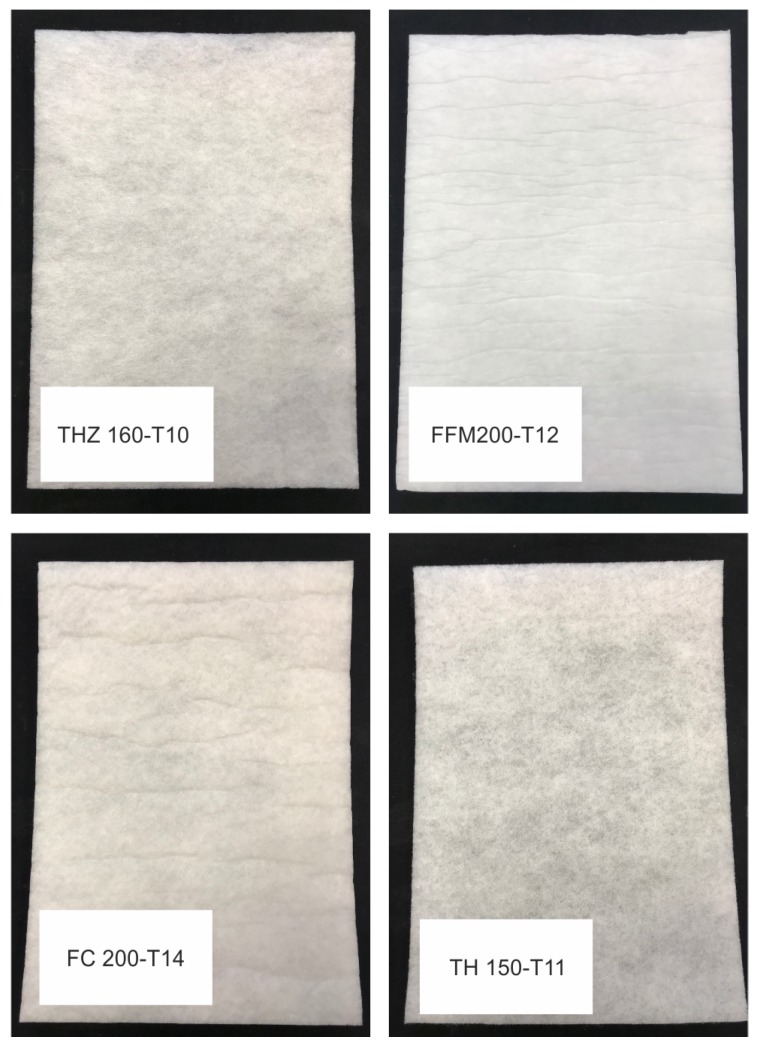
Photographs of four different fleece samples used in this study.

Fiber reinforcement of aerogels has been investigated within the past two decades. Parmenter and Milstein [15] used glass fibers to reinforce silica aerogels and investigated the mechanical and thermal properties of the resulting composites. Since then, several studies [16,17,18,19,20,21] have reported on such reinforcement procedures. This technique has been proven to be useful to enhance the properties of aerogels. However, to the best of our understanding, fiber reinforcement of super flexible silica aerogels has not been reported in literature thus far. Based on a synthesis procedure published in 2009 and 2011 [22,23,24], we developed a method to synthesize a fiber-reinforced material infiltrated with a super flexible silica-based aerogel. Four different fiber-reinforced flexible silica aerogels were investigated. The following polyester fibers (see Figure 1) were used; TWE Bocholt FFM 200-T12, TH 150-T11, THZ 160-T10, and FC 200-T14. Here, the nomenclature is as follows; for example, FFM 200-T12 stands for a fiber sheet as obtained from TWE Bocholt named FFM, having a grammage of 200 g m^−2^ and a thickness of 12 mm. For ease of use and reference, the four fibers mentioned above will be henceforth referred to as FFM, TH, THZ, and FC, respectively. In this manuscript, the morphology of the four composites was characterized by means of scanning electron microscopy (SEM). Furthermore, the mechanical behavior of all the four types of fiber-reinforced aerogels was investigated under uniaxial quasi-static compression. Keeping in mind aircraft applications, the mechanical response under different temperatures was studied. Cyclic tests with stepwise increasing amplitude reveal many interesting inelastic features of these aerogels.

The paper is organized as follows. Section 2 describes the synthesis procedure used to prepare the four kinds of fiber-reinforced aerogels; it also outlines the different characterization tools used to investigate the structural and mechanical properties of the aerogels. Section 3 presents the results, which are then carefully discussed.

## 2. Materials and Methods

In a typical synthesis approach, deionized water, urea, acetic acid, methyltrimethoxysilane (MTMS), dimethyldimethoxysilane (DMDMS), and a 25 wt% solution of cetyltrimethylammonium chloride (CTAC) are mixed in a weight ratio of 1:0.42:4.5 × 10^−4^:0.24:1.4 × 10^−2^:0.33, respectively. All chemicals are used as received without further purification. Primarily, urea is dissolved in water under vigorous stirring at 50 °C and under the addition of acetic acid. MTMS and CTAC solutions are subsequently added after complete dissolution of urea and the solution is further homogenized at 50 °C. DMDMS is added to the solution which is then stirred for 45 min at 50 °C. For more details, see [25].

The respective fibers were placed inside the molds and covered with the prepared solution in the ratio as shown in Table 1. The final composite is obtained after 24 h at 80 °C by washing with deionized water and drying under ambient conditions. The respective fiber mats only indicate the high versatility of our approach in combining the aerogel compound with different fiber mats of different grammages or thicknesses.

**Figure 2:**
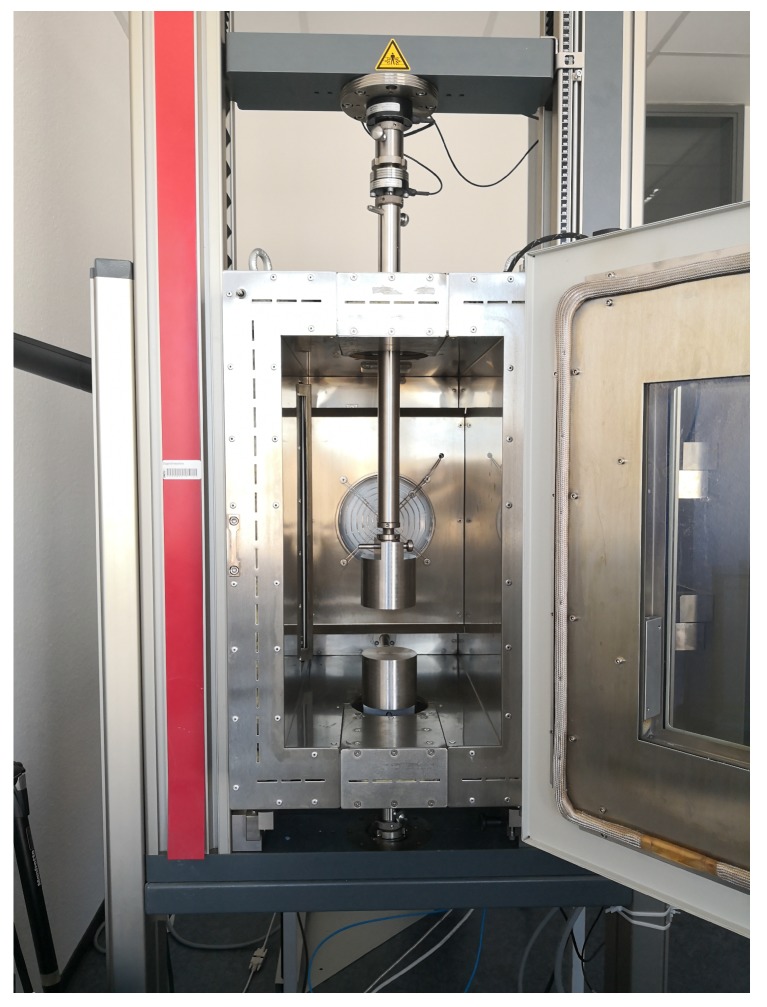
Thermal chamber with the uniaxial compression test setup.

For SEM micrographs the samples have been prepared on a carbon pad (Plano GmbH, Wetzlar, Germany) and sputter coated with platinum for 90 s at a pressure of 4 × 10^−2^ mbar (SCD 500, Baltec, Pfäffikon, Switzerland). The coated samples have been transferred into the SEM (Ultra 55, Zeiss, Oberkochen, Germany), and micrographs have been recorded using 2 kV acceleration voltage and a working distance of 3.5 mm. The thermal conductivity of all the samples was measured using a heat flow meter (HFM) 436 Lambda, Netzsch, Germany, at atmospheric pressure and under different temperatures between 0 °C and 60 °C. The specimen geometry of 12 mm × 12 mm × T mm, where T is the thickness of the fiber mat as discussed in Section 1, was chosen. For testing their mechanical behavior, all the four types of fiber-reinforced aerogels were subjected to uniaxial quasi-static compression. A universal testing machine (UTM) Z010 and a thermal chamber BW91271, both, from Zwick/Roell GmbH & Co. KG, Ulm, Germany were used. The setup is displayed in Figure 2.

**Figure 3:**
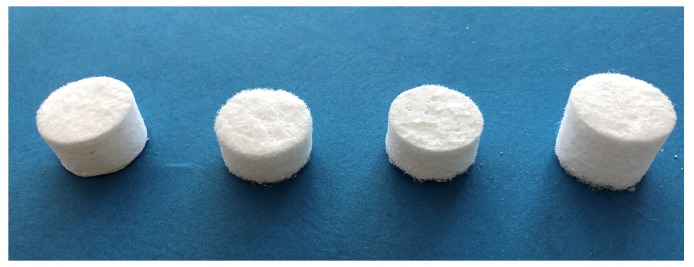
Photograph of the four different aerogel composites based on the following fiber mats. Left to right: FFM, TH, THZ, and FC.

The thermal chamber has heating capabilities up to 250 °C, while for cooling to lower temperatures (up to −80 °C), liquid nitrogen, purchased from Wagner GmbH, Eschweiler, Germany, was used. All the aerogel specimens had a cylindrical form with a diameter of 20 mm (see Figure 3). All the compression tests were conducted at three temperatures: 50 °C, 0 °C and −50 °C. A load cell of 1 kN was used. To assure isothermal conditions, a dwell time of 30 min was considered for reaching a constant temperature within the chamber after every change of the specimen. Furthermore, uniaxial tension tests on bar-shaped specimens (dimensions: 40 mm × 15 mm × T mm) of pure flexible aerogel, the four types of pure fiber mats and the four types of fiber-reinforced composites were carried out on the UTM by Latzke, Wiehl, Germany, with a load cell of 1 kN. The tension tests were conducted at a controlled room temperature of 20 °C. All tension and compression tests were conducted under a strain rate of 10%/min.

## 3. Results and Discussion

Figure 3 shows the macroscopic appearance of the four composite materials tested. In all four cases, the aerogels could successfully be synthesized regardless of the grammage or absolute height of the fiber mat. The surface morphology of the fibers predefines that of the composite because the structure of the fibers dominates over that of the aerogel after synthesis. All four systems can be shaped after the synthesis process and cut.

**Figure 4:**
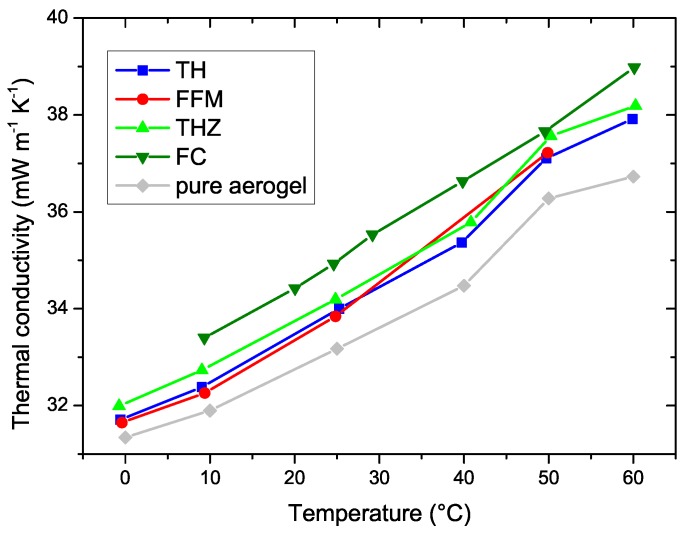
HFM-based thermal conductivity measurement results for the four aerogel composites and pure flexible silica aerogel under different temperatures.

**Figure 5:**
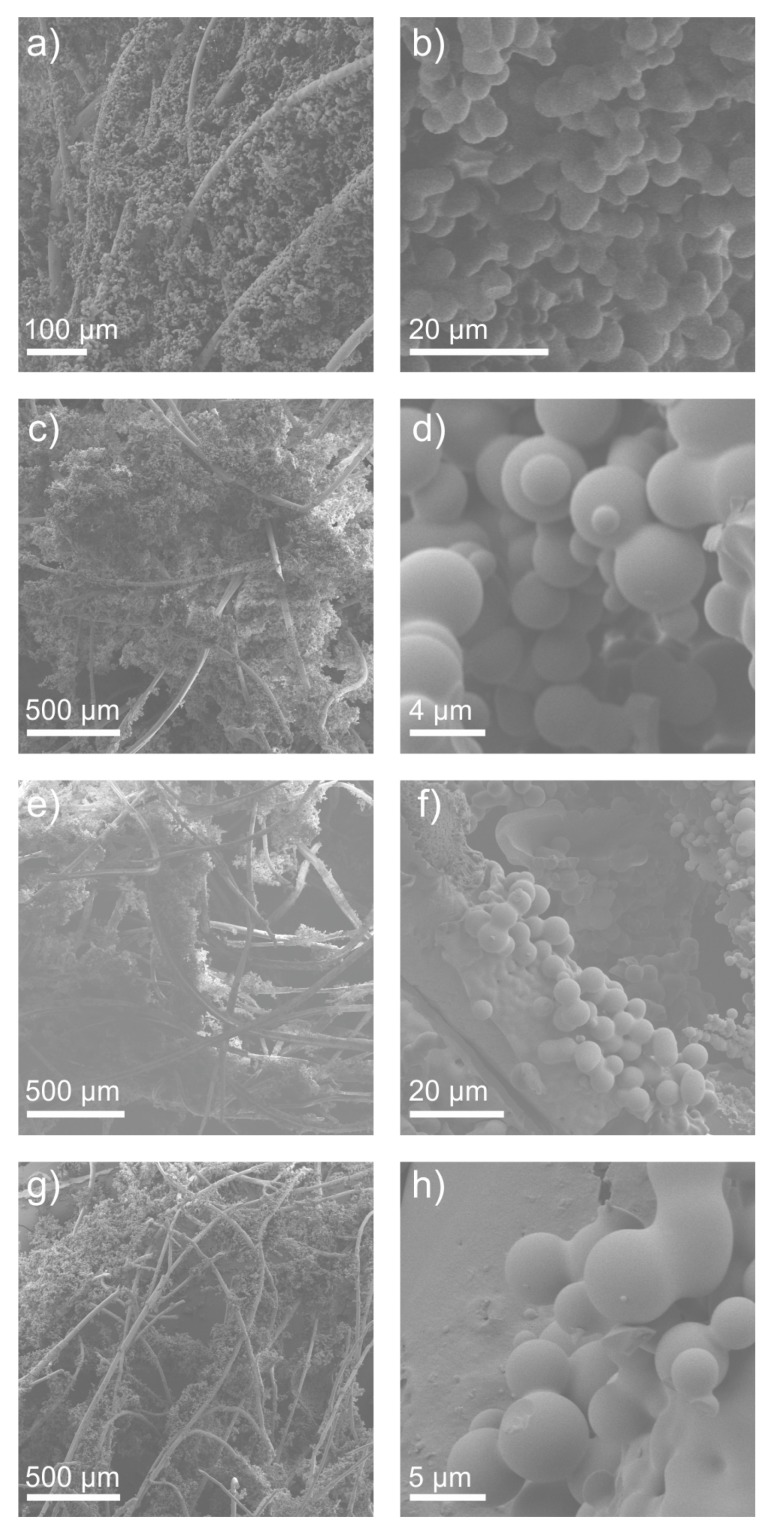
Scanning electron micrographs of aerogel composites: (**a**,**b**) FFM, (**c**,**d**) TH, (**e**,**f**) THZ, and (**g**,**h**) FC.

Thermal conductivity measurements reveal that the macroscopic properties of the fiber fleece exhibit low influence on the thermal properties of the composites. In the temperature range of 0 to 60 °C, the thermal conductivity of the samples slightly increases from 31 mW m^−1^ K^−1^ to approximately 39 mW m^−1^ K^−1^, as displayed in Figure 4. Upon visualization, it is realized that the fiber reinforcement has no significant influence on the thermal conductivity. Although the grammage was 150 g m^−2^ in the one case and 200 g m^−2^ in the other, the thermal conductivity does not differ significantly. Therefore, we conclude that the fibers are completely covered and protected by the aerogel and do not exhibit direct influence on the thermal conductivity of the compound, at least for the selected grammages. It can also be expected that very high grammages will exhibit an influence on the performance as the heat transfer paths might not be covered by the aerogel but only by the fiber material. Scanning electron micrographs reveal the inner structure of the composites (see Figure 5). The globular structure of the primary particles of the aerogels is preserved in all four different samples. Also the distribution of aerogel within the fiber network is homogeneous and independent of the chosen fibers. Good contact between the aerogel and the fibers could be established. The diameter of the fibers differs in the range of 20 to 35 µm. The smallest fiber diameters could be found in FFM. The envelope density $\rho_{e}$ was measured by the ratio of the specimen’s mass and defined volume. The skeletal density $\rho_{s}$ of the samples was measured by helium pycnometry with an AccuPyc (Micromeritics). Accordingly, the porosity, *P*, was calculated by the following formula

(1) $$P=1-\frac{\rho_{e}}{\rho_{s}}.$$

The porosity of all the four aerogel composites was measured to be approximately 95%. This value is similar to that observed for pure flexible aerogels under consideration [26], which shows that fiber reinforcement in the 1:1 fiber to sol volume ratio has negligible influence on porosity.

**Figure 6:**
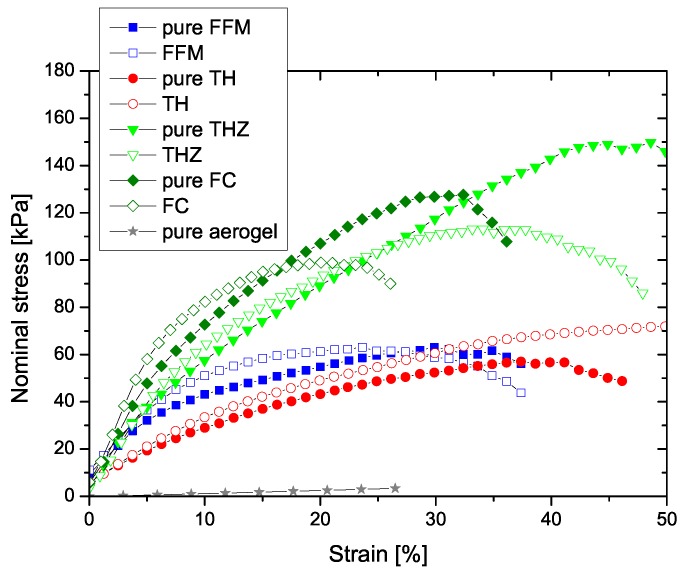
Comparison of the uniaxial tensile response of the four aerogel composites (FFM, TH, THZ, and FC) with their pristine components: pure fiber mats (pure FFM, pure TH, pure THZ, and pure FC) and pure silica aerogel.

Figure 6 shows a comparison of the responses of each of the four fiber-reinforced aerogel composites with their pristine components, under uniaxial quasi-static tension. It is seen that the pure flexible silica aerogel demonstrates very weak mechanical strength in comparison to the pure fiber mats. However, after reinforcement, with a 1:1 ratio of the individual components, the composite shows enhanced strength while retaining very low thermal conductivities as discussed earlier. While the stiffness of the composite is much higher than that of the pure aerogel, it is marginally higher than that of their respective pure fiber mats. Note also that the failure strain of the composites is smaller than that of the pure fiber mats, which shows the influence of the aerogel matrix. Of special interest was the temperature dependency of the composites on their mechanical behavior and damage. To study this dependency, uniaxial quasi-static compression tests were conducted at three different temperatures: 50 °C, 0 °C, and −50 °C.

**Figure 7:**
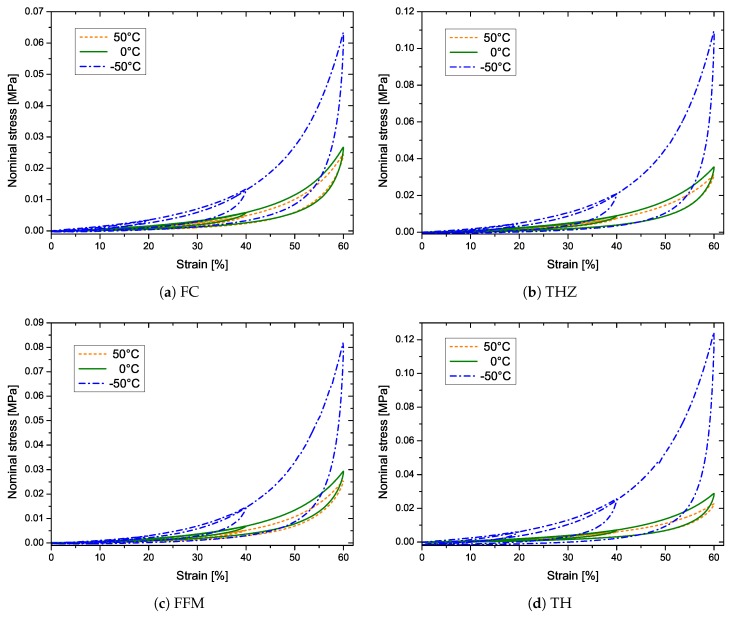
Stress–strain curves under cyclic compression with stepwise increasing strain amplitude of 20%, 40%, and 60%: (**a**) FC, (**b**) THZ, (**c**) FFM, and (**d**) TH. For an interested reader, the detailed individual curves along with insets are provided in the Supplementary Materials (see Figures S1–S3, S7–S9, S13–S15, S19–S21, respectively).

First, the aerogel composites were subjected to cyclic compression with step-wise increasing strain amplitude (see Figure 7). For an interested reader, the detailed individual curves along with insets are provided in the Supplementary Materials (see Figures S1–S3, S7–S9, S13–S15, S19–S21). The following strain amplitudes were applied; 20%, 40%, and 60%. The aerogels exhibit inelastic effects such as cyclic stress-softening, hysteresis, and permanent set (residual deformation). Interestingly, these aerogels exhibit an internal memory. This means that when the compression exceeds the previously applied maximal strain, the stress–strain curve returns to the monotonous stress–strain curve (primary curve), just like it would be if monotonically loaded. This effect is known from rubbers and referred to as the Mullins effect [27]. Furthermore, the amount of softening and hysteresis strongly increase with increasing maximal strain. Similar effects are observed in all the temperature environments, but with different magnitudes. All the four types of fiber-reinforced aerogels, exhibit stiffening of their stress–strain response at lower temperatures. The amount of stiffening is marginal when the surrounding temperature is reduced to 0 °C from 50 °C. However, very strong stiffening (up to four times) is observed by cooling down to −50 °C. THZ and FC show the most pronounced stiffening at −50 °C, followed by TH and FFM. Hysteresis represents the amount of energy dissipated during the cyclic deformation. Similar to the stiffening behavior, an increase in the amount of dissipated energy is observed with decreasing temperature. At 50 °C as well as at 0 °C the amount of energy dissipated is almost similar, while at −50 °C it is very pronounced. Moreover, the aerogels were also subjected to a compression of 80% strain, where each cycle was repeated three times. All the individual curves are provided in the Supplementary Materials (see Figures S4–S6, S10–S12, S16–S18, S22–S24).

Under compression, native silica aerogels as well as many other aerogel composites exhibit three regimes: (a) linear elastic, (b) plateau, and (c) densification [28,29,30]. In the case of the four composites tested here, only a linear elastic regime (strains <10%), and the hardening beyond it, can be observed. One of the primary reasons behind this mechanical behavior could be the super flexibile nature of the composites. Both, the aerogel and fiber phases are very flexible, such that the fiber matrices collapse only due to elastic buckling. Moreover, there seems to be a smooth transition between the buckling of fibers and the subsequent densification of the network. The aerogels show a much less permanent set (<10% residual strain upon 80% compression) at 50 °C, as well as at 0 °C, as the specimens almost reversed back to their original position when unloaded. The elastic nature however is compromised at −50 °C, where the permanent set is quite pronounced. It is the most prominent in the case of TH, while it is the least for FFM.

## 4. Conclusions

In this paper, the synthesis and characterization of flexible silica aerogels reinforced with four different types of fibers is presented. To this end, first, thermal properties were investigated. Here, the thermal conductivity of the pure silica aerogels is marginally influenced by fiber reinforcement. All four composites demonstrated thermal conductivity similar to that of pure silica aerogels. This is attributed to the formation of a silica aerogel coating around the fibers. Porosity of approximately 95% was observed in all cases, which shows that fiber reinforcement, in our case considering a 1:1 fiber-to-sol volume ratio, does not influence the porosity. Although thermal conductivity was retained, mechanical strength significantly increased after fiber reinforcement in all cases (up to 30 times). This is because of the high strength of the pristine fiber mats. Furthermore, the temperature-dependent mechanical behavior and properties under cyclic uniaxial compression are also presented. All the four types of fiber-reinforced aerogels exhibit inelastic phenomena such as hysteresis, stress-softening, and residual deformation. It can be concluded that the cooler the environmental conditions, the stiffer the mechanical response of the composites. The stiffening is highly pronounced under sub-zero temperatures, in some cases up to four times. Upon cooling from 50 °C to 0 °C, there is no significant change to the flexibility of the composite. A substantial residual deformation appears under an operating temperature of −50 °C. The goal of this study was to show that while the thermal performance of the super flexible aerogel composites does not significantly change upon composite formation, it results in a much enhanced, stable mechanical performance under a wide temperature range.

## Figures and Tables

**Table 1:** Ratio of the fiber volume to the sol volume.

Fiber Type	Fiber Volume (cm^−3^)	Sol Volume (cm^−3^)
FFM	20.5 × 27 × 1.2	665
TH	20.5 × 27 × 1.1	610
THZ	20.5 × 27 × 1.0	555
FC	20.5 × 27 × 1.4	775

## Supplementary Materials

The following are available online at https://www.mdpi.com/1996-1944/12/18/2878/s1, Figure S1: Stress-strain curves of FC under cyclic compression with stepwise increasing strain amplitude of 20%, 40% and 60% at 50 °C, Figure S2: Stress-strain curves of FC under cyclic compression with stepwise increasing strain amplitude of 20%, 40% and 60% at 0 °C, Figure S3: Stress-strain curves of FC under cyclic compression with stepwise increasing strain amplitude of 20%, 40% and 60% at −50 °C, Figure S4:Stress-strain curves of FC from cyclic compression up to 80% strain, where every cycle was repeated three times, at 50 °C, Figure S5: Stress-strain curves of FC from cyclic compression up to 80% strain, where every cycle was repeated three times, at 0 °C, Figure S6: Stress-strain curves of FC from cyclic compression up to 80% strain, where every cycle was repeated three times, at −50 °C, Figure S7: Stress-strain curves of THZ under cyclic compression with stepwise increasing strain amplitude of 20%, 40% and 60% at 50 °C, Figure S8: Stress-strain curves of THZ under cyclic compression with stepwise increasing strain amplitude of 20%, 40% and 60% at 0 °C, Figure S9: Stress-strain curves of THZ under cyclic compression with stepwise increasing strain amplitude of 20%, 40% and 60% at −50 °C, Figure S10: Stress-strain curves of THZ from cyclic compression up to 80% strain, where every cycle was repeated three times, at 50 °C, Figure S11: Stress-strain curves of THZ from cyclic compression up to 80% strain, where every cycle was repeated three times, at 0 °C, Figure S12: Stress-strain curves of THZ from cyclic compression up to 80% strain, where every cycle was repeated three times, at −50 °C, Figure S13: Stress-strain curves of FFM under cyclic compression with stepwise increasing strain amplitude of 20%, 40% and 60% at 50 °C, Figure S14: Stress-strain curves of FFM under cyclic compression with stepwise increasing strain amplitude of 20%, 40% and 60% at 0 °C, Figure S15: Stress-strain curves of FFM under cyclic compression with stepwise increasing strain amplitude of 20%, 40% and 60% at −50 °C, Figure S16: Stress-strain curves of FFM from cyclic compression up to 80% strain, where every cycle was repeated three times, at 50 °C, Figure S17: Stress-strain curves of FFM from cyclic compression up to 80% strain, where every cycle was repeated three times, at 0 °C, Figure S18: Stress-strain curves of FFM from cyclic compression up to 80% strain, where every cycle was repeated three times, at 50 °C, Figure S19: Stress-strain curves of TH under cyclic compression with stepwise increasing strain amplitude of 20%, 40% and 60% at 50 °C, Figure S20: Stress-strain curves of TH under cyclic compression with stepwise increasing strain amplitude of 20%, 40% and 60% at 0 °C, Figure S21: Stress-strain curves of TH under cyclic compression with stepwise increasing strain amplitude of 20%, 40% and 60% at −50 °C, Figure S22: Stress-strain curves of TH from cyclic compression up to 80% strain, where every cycle was repeated three times, at 50 °C, Figure S23: Stress-strain curves of TH from cyclic compression up to 80% strain, where every cycle was repeated three times, at 0 °C, Figure S24: Stress-strain curves of TH from cyclic compression up to 80% strain, where every cycle was repeated three times, at −50 °C.

## Abbreviations

The following abbreviations are used in this manuscript.

EPS	expanded polystyrene
SEM	scanning electron microscopy
MTMS	methyltrimethoxysilane
DMDS	dimethyldimethoxysilane
CTAC	cetyltrimethylammonium chloride
HFM	heat flow meter
