# Supplementary material for: Temperature-Dependent Stiffening and Inelastic Behavior of Newly Synthesized Fiber-Reinforced Super Flexible Silica Aerogels
Source: Materials (Basel). 2019 Sep 6;12(18):2878. doi: 10.3390/ma12182878 (PMC6766033; doi:10.3390/ma12182878)
Supplement: Supplementary file 1 [file materials-12-02878-s001.pdf]

Supplementary Materials: Temperature-Dependent Stiffening and Inelastic Behavior of Newly Synthesized Fiber-Reinforced Super Flexible Silica Aerogels

Ameya Rege [1,†,*], Pascal Voepel [1,†,*], Emrah Okumus [1,2], Markus Hillgärtner [2], Mikhail Itskov [2] and Barbara Milow [1]

The following supplementary material provides individual compressive stress-strain curves for each type of fiber-reinforced aerogel (FC, THZ, FFM, TH) at each temperature condition (50°C, 0°C and -50°C). Stress strain curves provided are of the following two kinds: under cyclic compression with stepwise increasing strain amplitude of 20%, 40% and 60%, and from cyclic compression up to 80% strain, where every cycle was repeated three times.

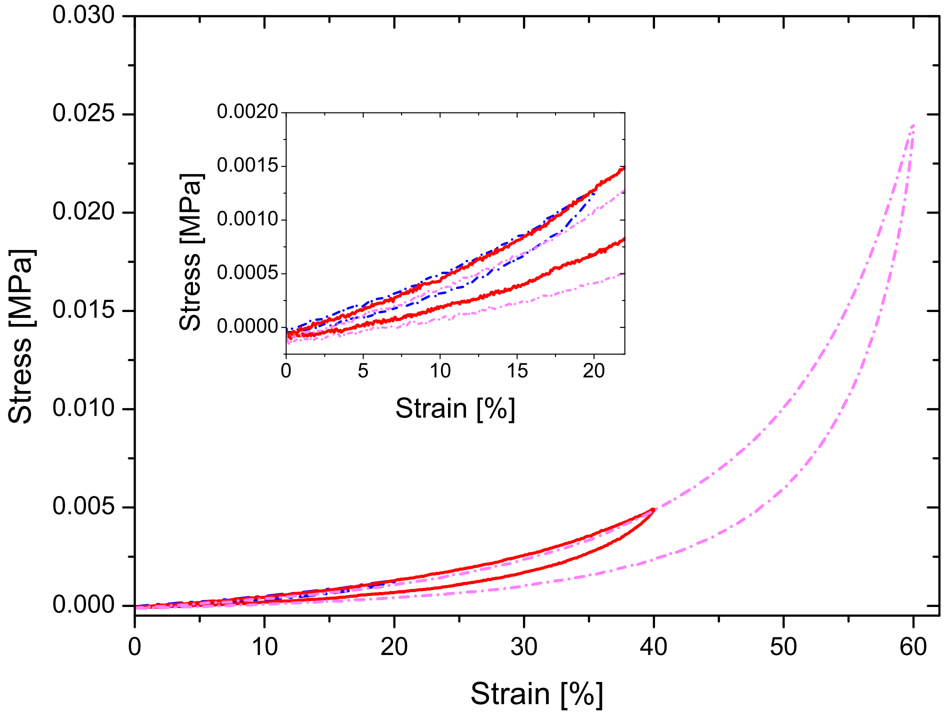

Figure S1. Stress-strain curves of FC under cyclic compression with stepwise increasing strain amplitude of 20%, 40% and 60% at 50°C

Materials **2019**, *xx* ; doi:10.3390/maxx010005

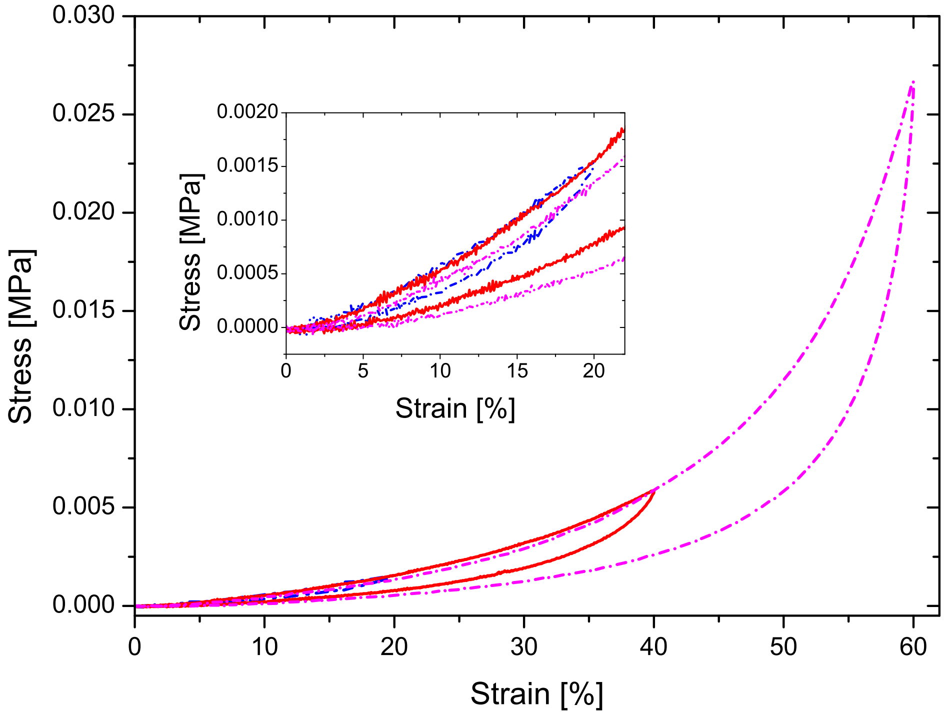

Figure S2. Stress-strain curves of FC under cyclic compression with stepwise increasing strain amplitude of 20%, 40% and 60% at 0°C

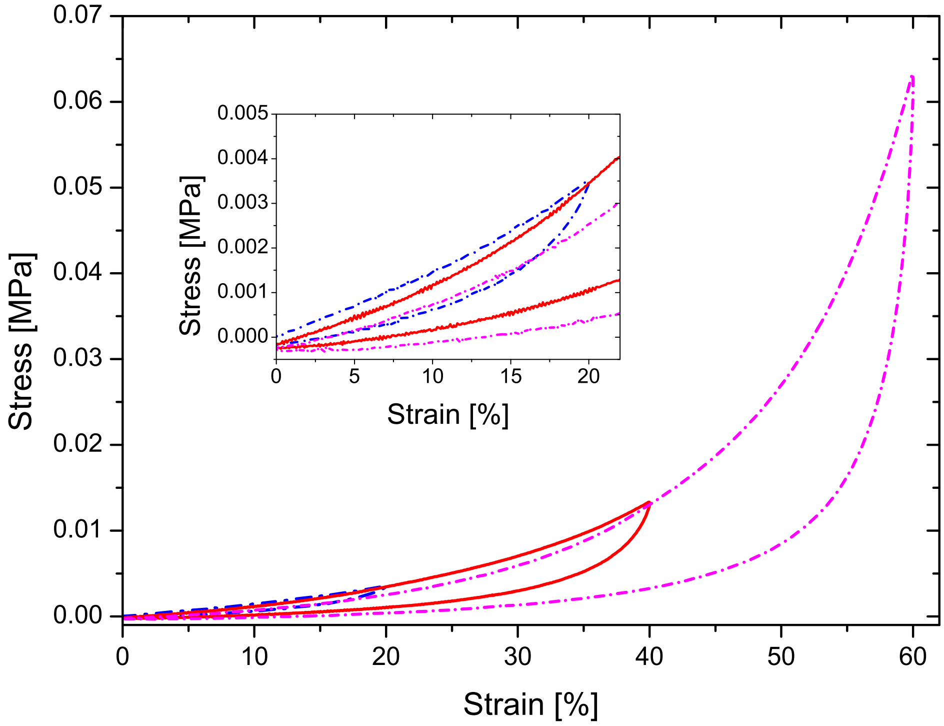

Figure S3. Stress-strain curves of FC under cyclic compression with stepwise increasing strain amplitude of 20%, 40% and 60% at -50°C

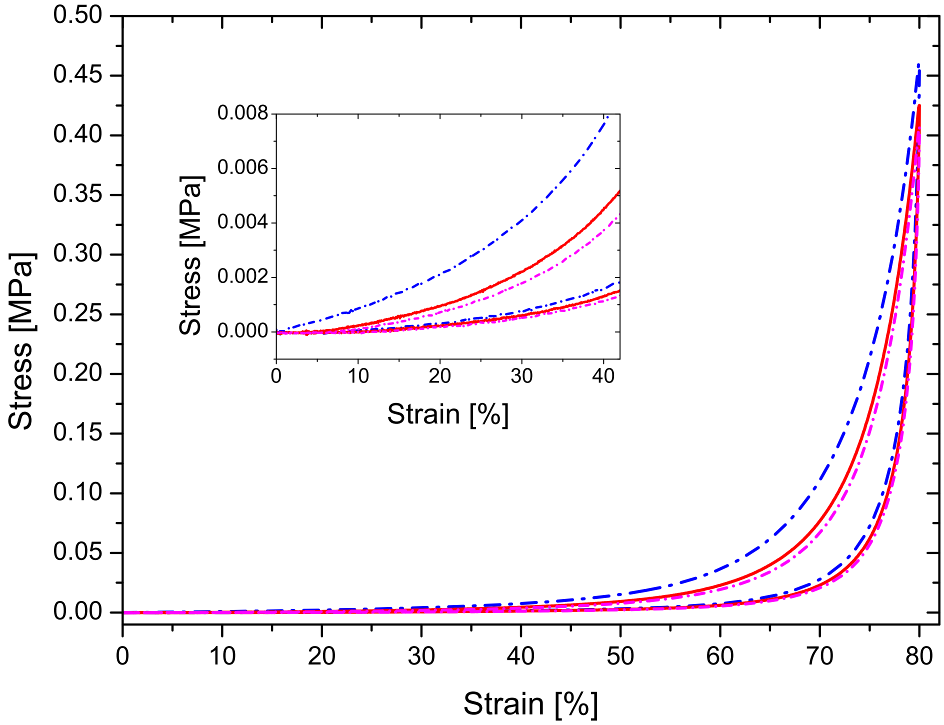

Figure S4. Stress-strain curves of FC from cyclic compression up to 80% strain, where every cycle was repeated three times, at 50°C

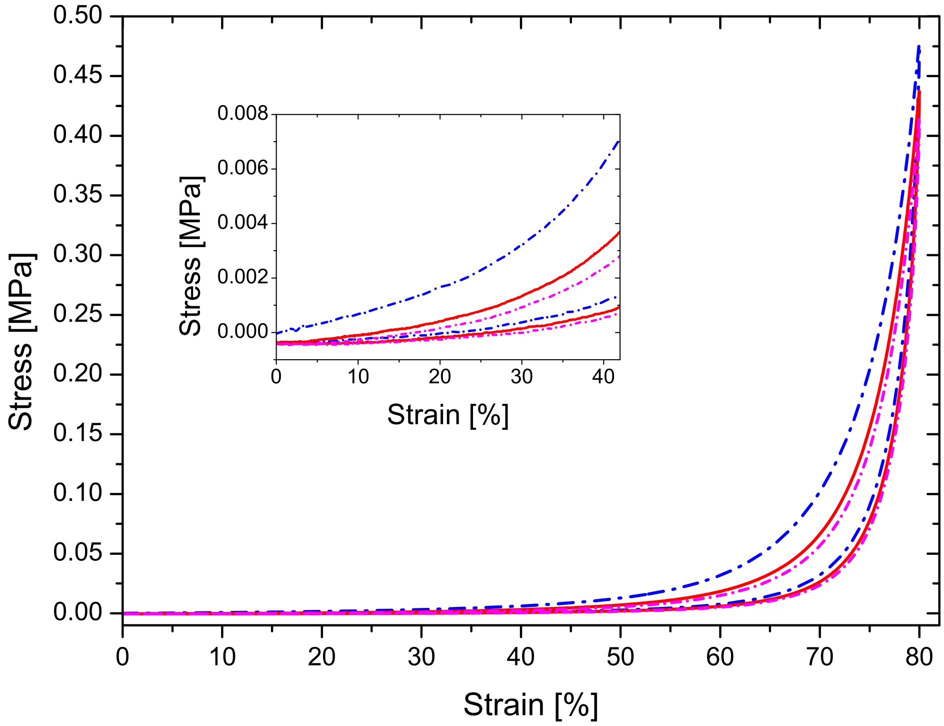

Figure S5. Stress-strain curves of FC from cyclic compression up to 80% strain, where every cycle was repeated three times, at 0°C

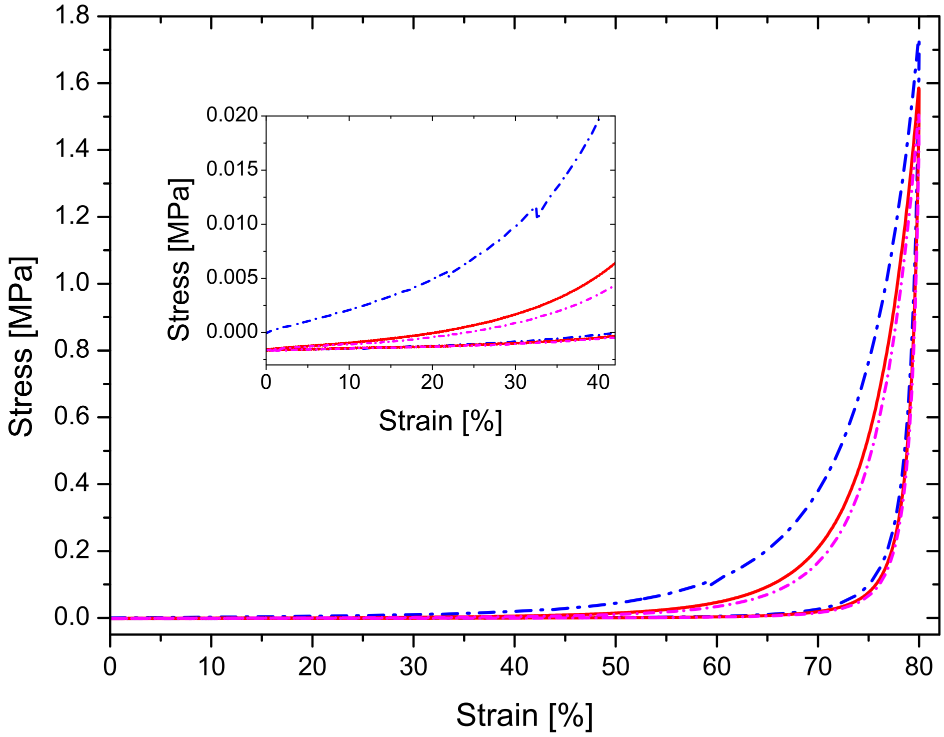

Figure S6. Stress-strain curves of FC from cyclic compression up to 80% strain, where every cycle was repeated three times, at -50°C

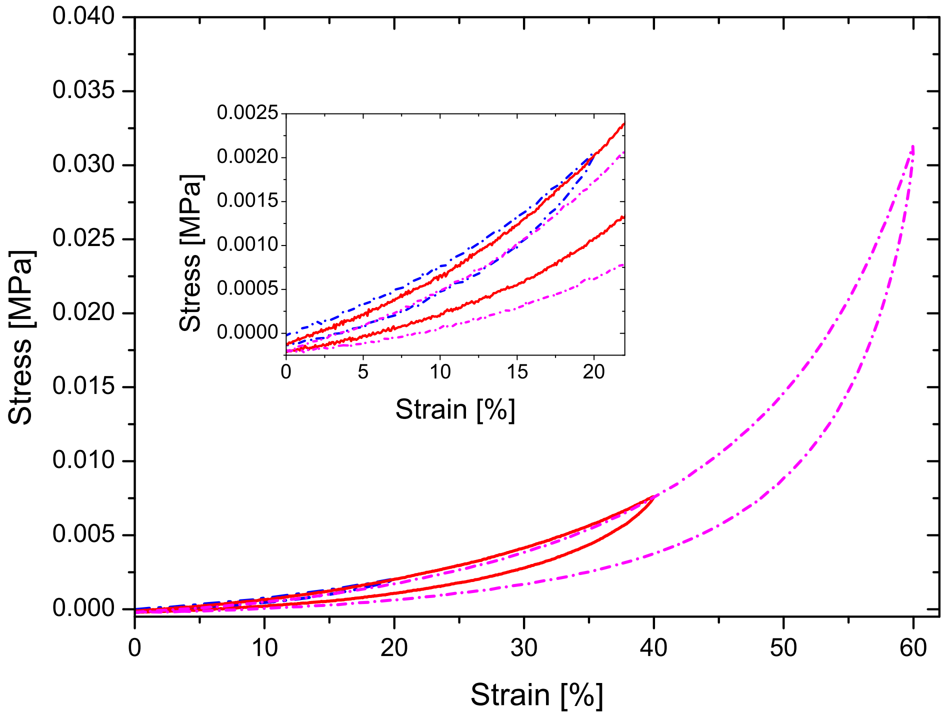

Figure S7. Stress-strain curves of THZ under cyclic compression with stepwise increasing strain amplitude of 20%, 40% and 60% at 50°C

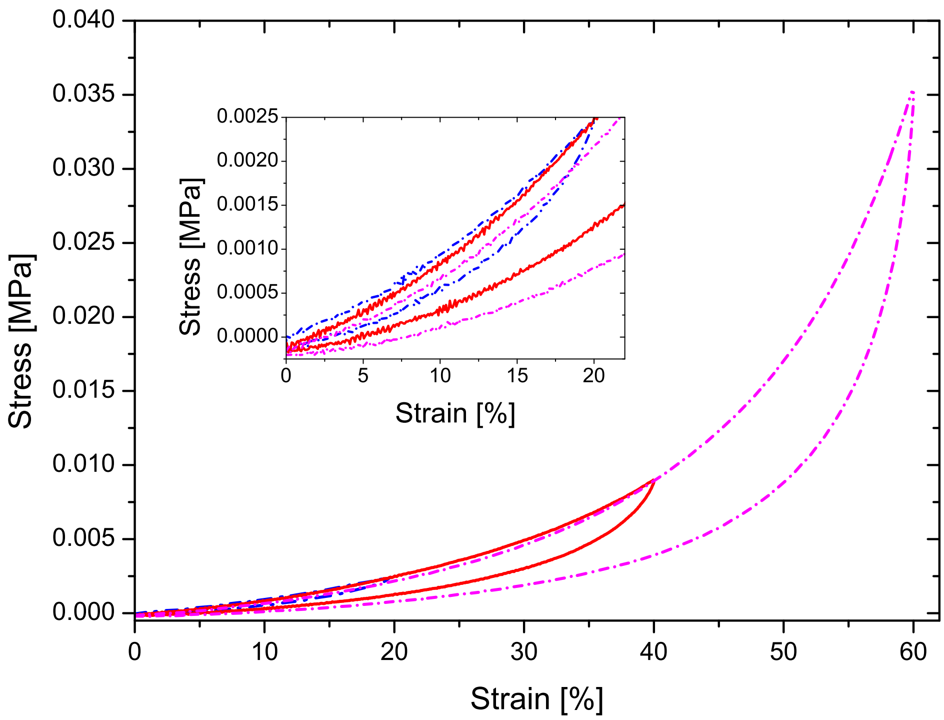

Figure S8. Stress-strain curves of THZ under cyclic compression with stepwise increasing strain amplitude of 20%, 40% and 60% at 0°C

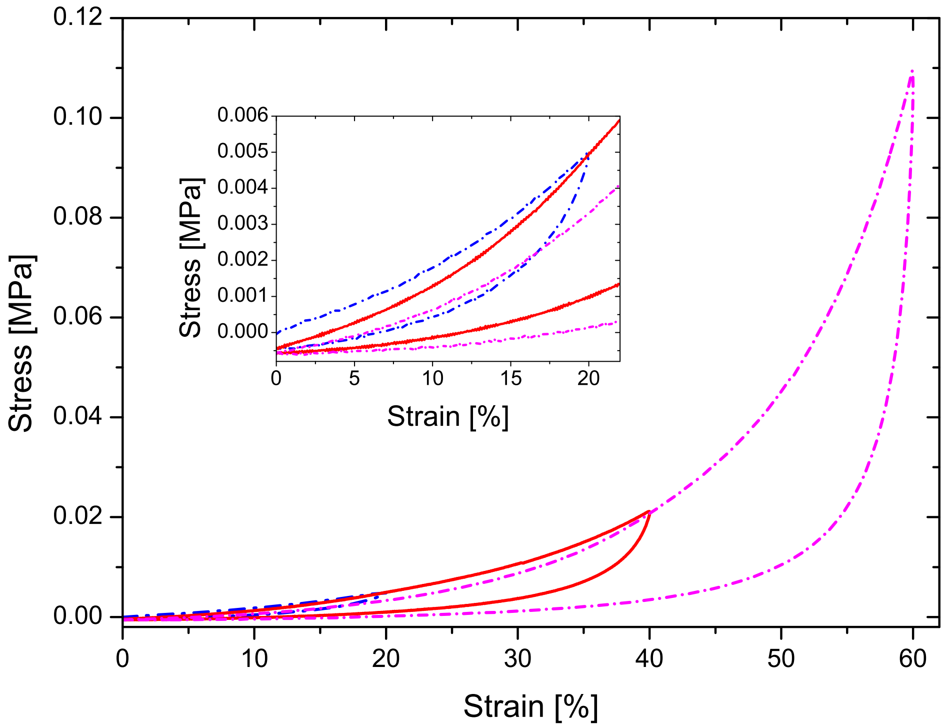

Figure S9. Stress-strain curves of THZ under cyclic compression with stepwise increasing strain amplitude of 20%, 40% and 60% at -50°C

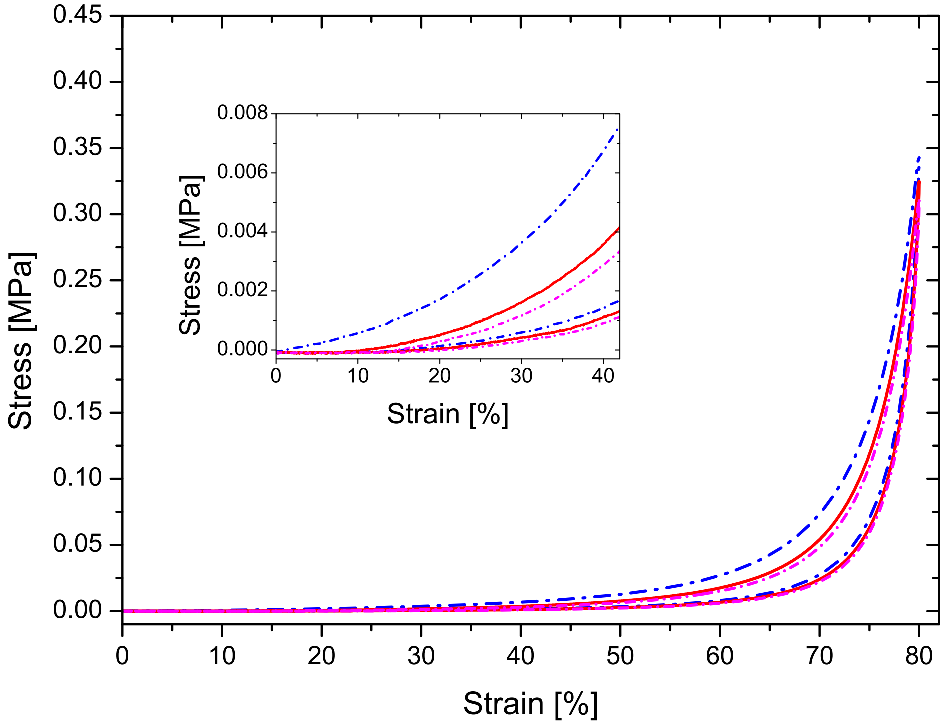

Figure S10. Stress-strain curves of THZ from cyclic compression up to 80% strain, where every cycle was repeated three times, at 50°C

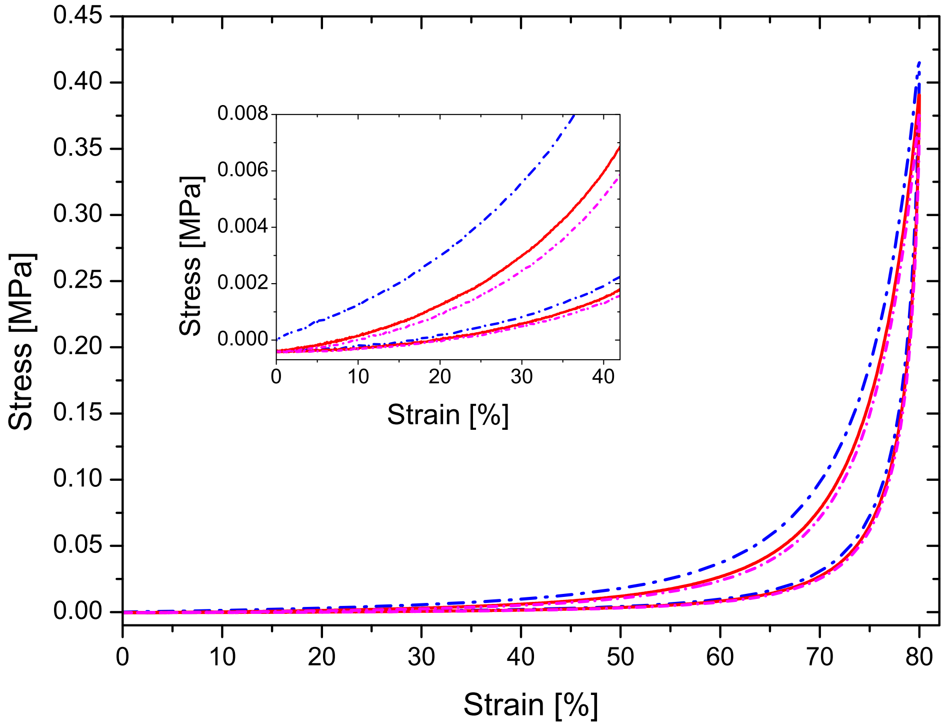

Figure S11. Stress-strain curves of THZ from cyclic compression up to 80% strain, where every cycle was repeated three times, at 0°C

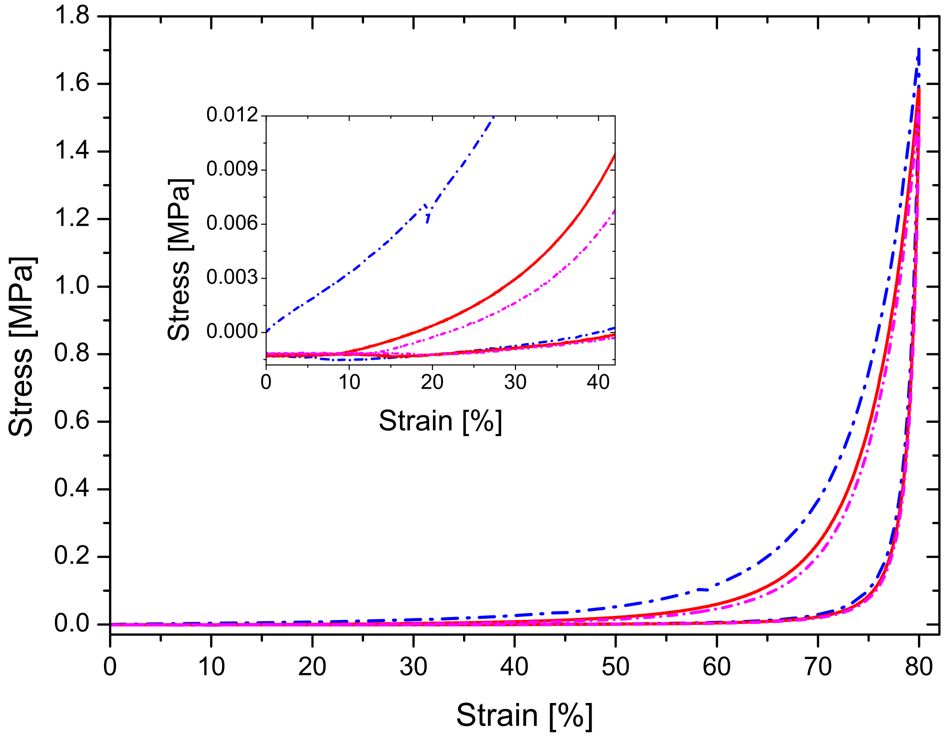

Figure S12. Stress-strain curves of THZ from cyclic compression up to 80% strain, where every cycle was repeated three times, at -50°C

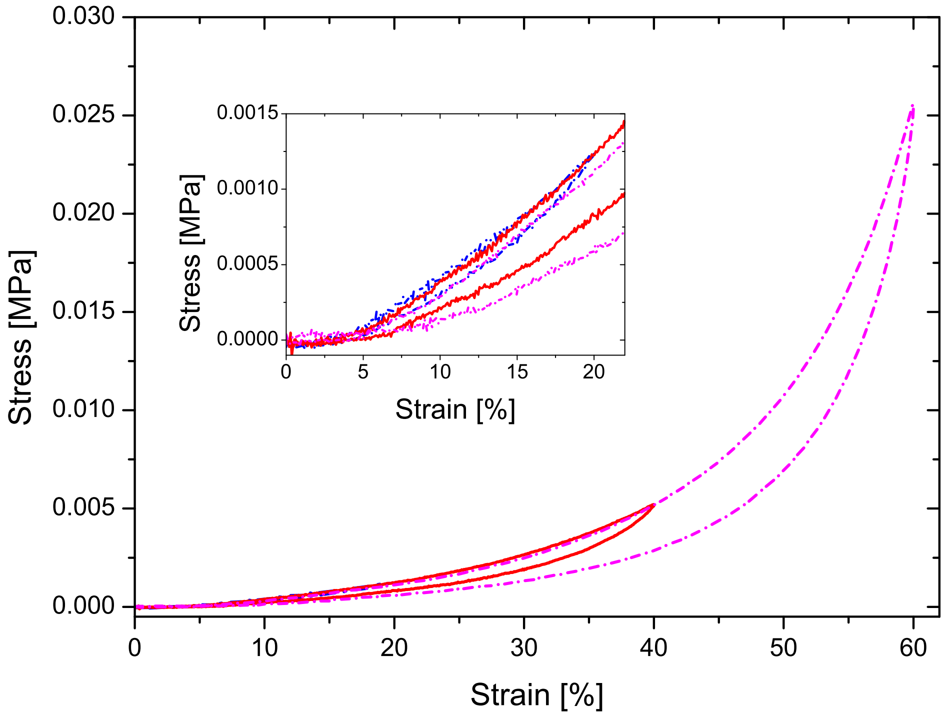

Figure S13. Stress-strain curves of FFM under cyclic compression with stepwise increasing strain amplitude of 20%, 40% and 60% at 50°C

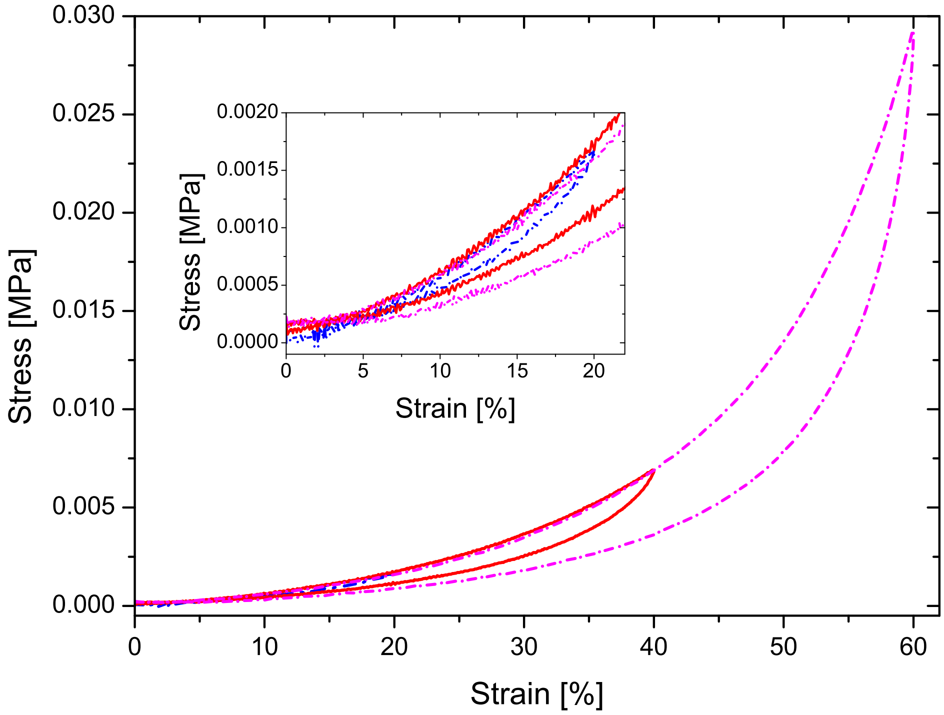

Figure S14. Stress-strain curves of FFM under cyclic compression with stepwise increasing strain amplitude of 20%, 40% and 60% at 0°C

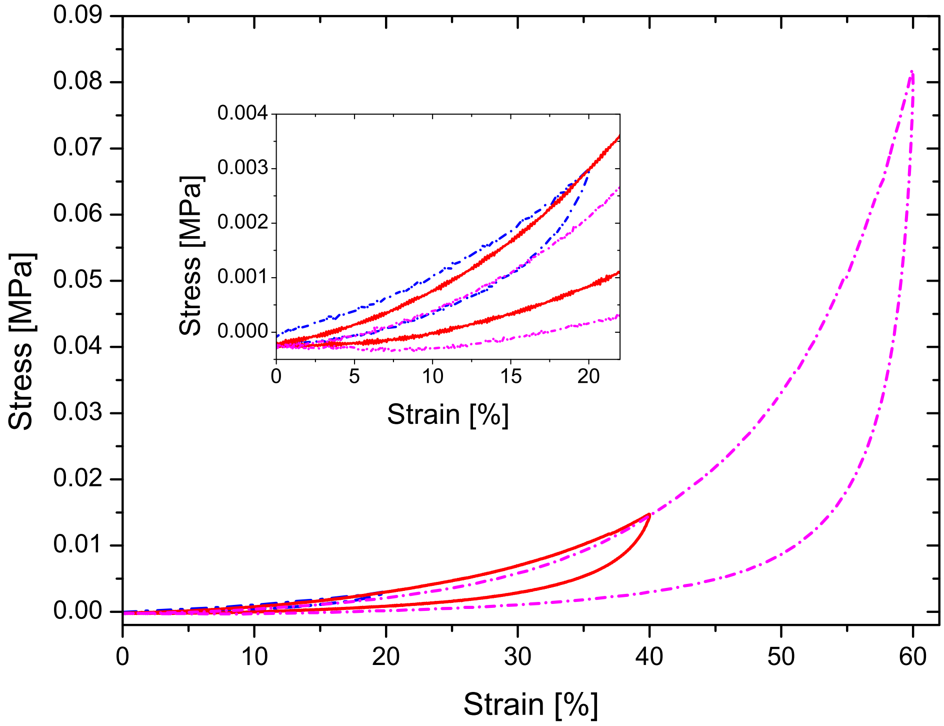

Figure S15. Stress-strain curves of FFM under cyclic compression with stepwise increasing strain amplitude of 20%, 40% and 60% at -50°C

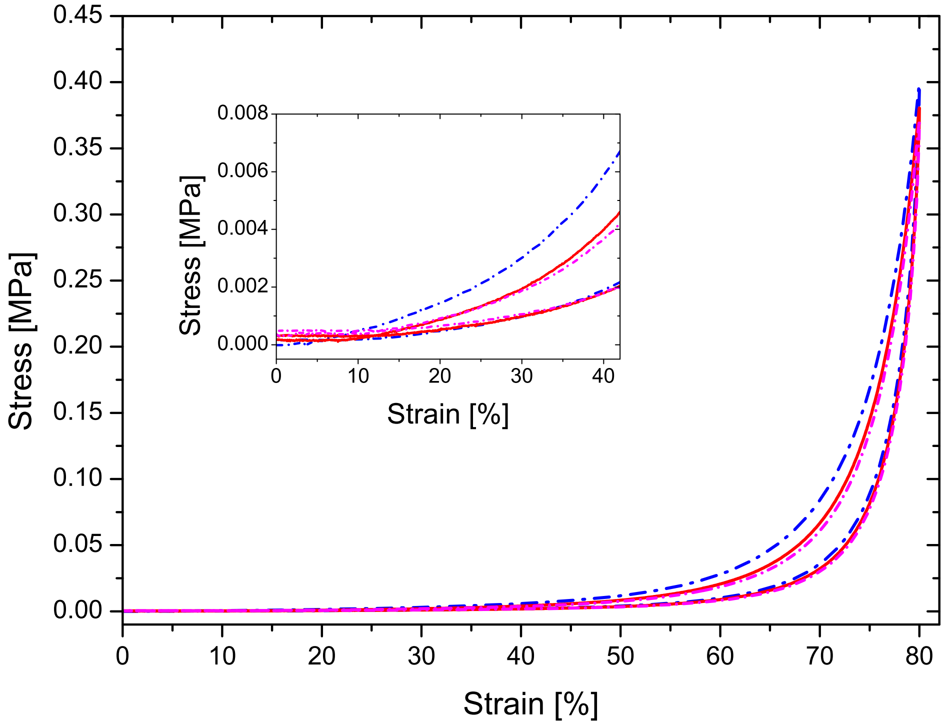

Figure S16. Stress-strain curves of FFM from cyclic compression up to 80% strain, where every cycle was repeated three times, at 50°C

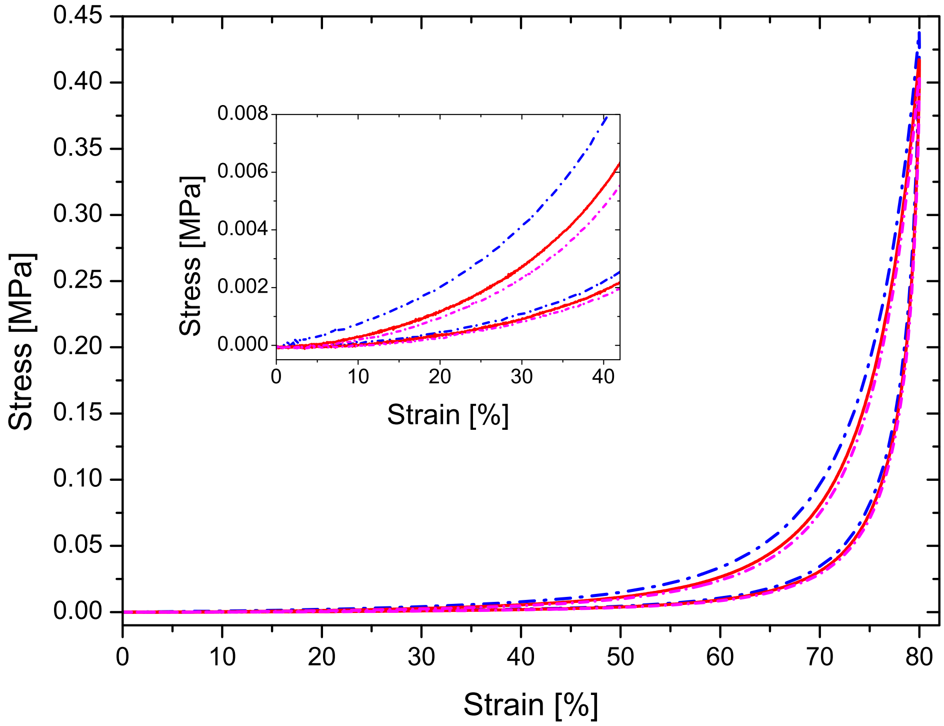

Figure S17. Stress-strain curves of FFM from cyclic compression up to 80% strain, where every cycle was repeated three times, at 0°C

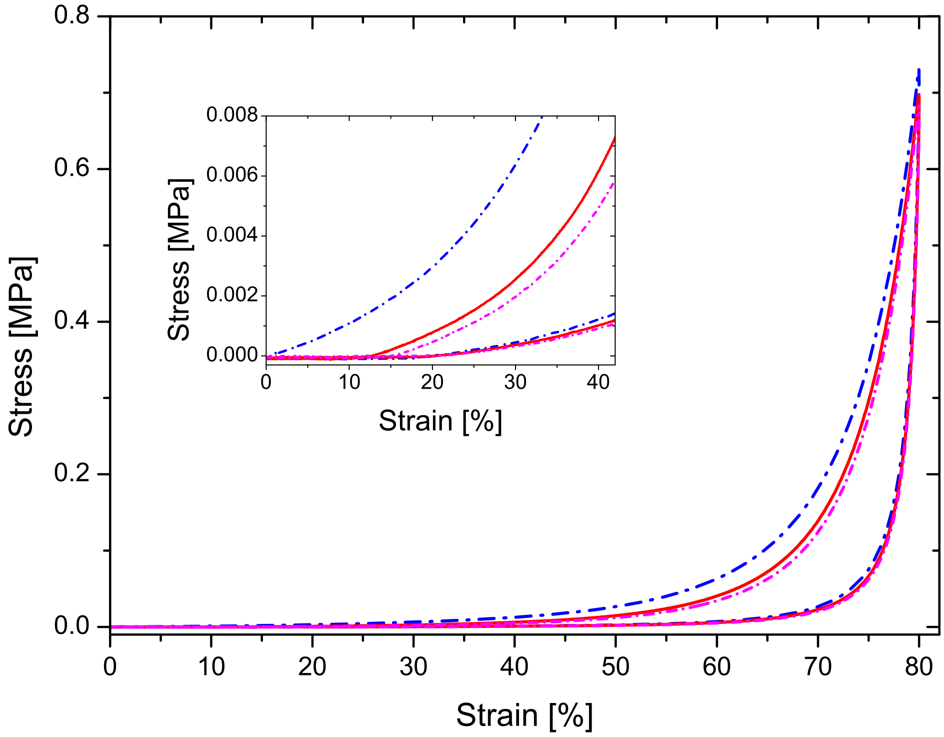

Figure S18. Stress-strain curves of FFM from cyclic compression up to 80% strain, where every cycle was repeated three times, at 50°C

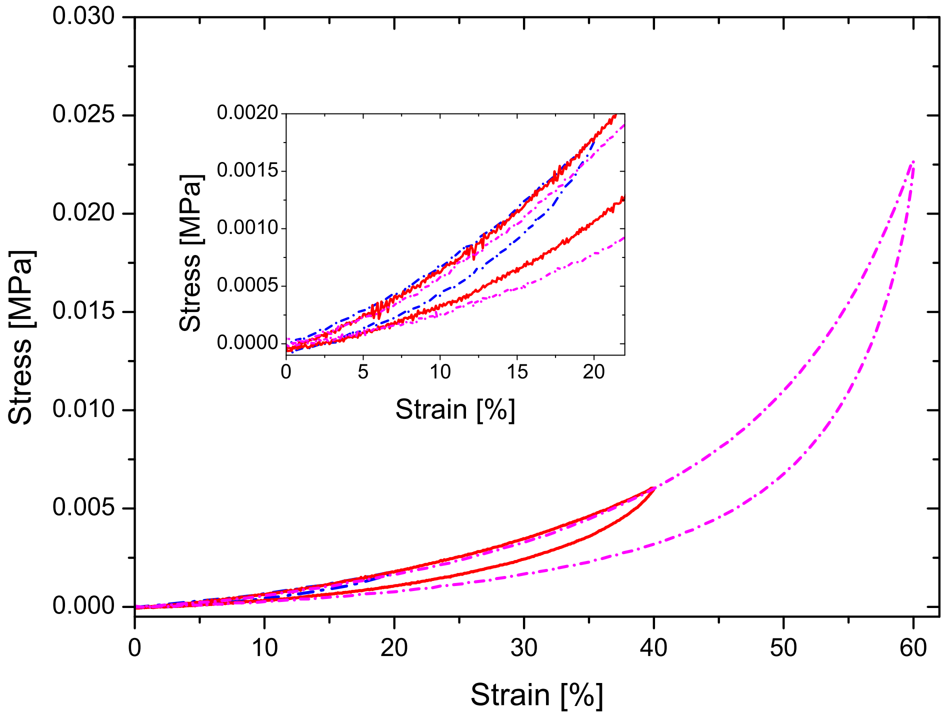

Figure S19. Stress-strain curves of TH under cyclic compression with stepwise increasing strain amplitude of 20%, 40% and 60% at 50°C

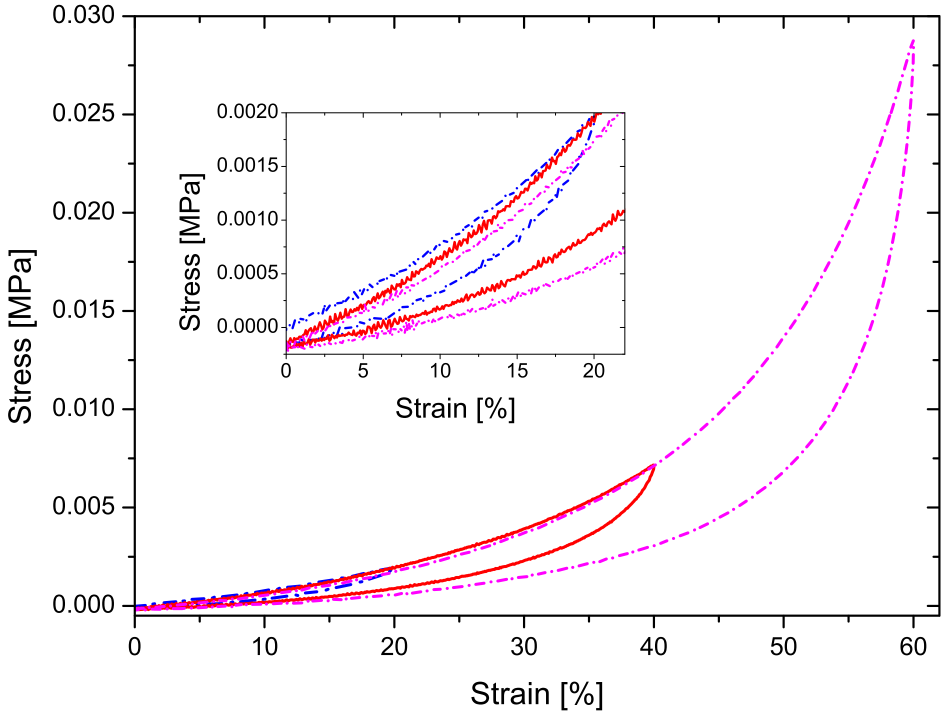

Figure S20. Stress-strain curves of TH under cyclic compression with stepwise increasing strain amplitude of 20%, 40% and 60% at 0°C

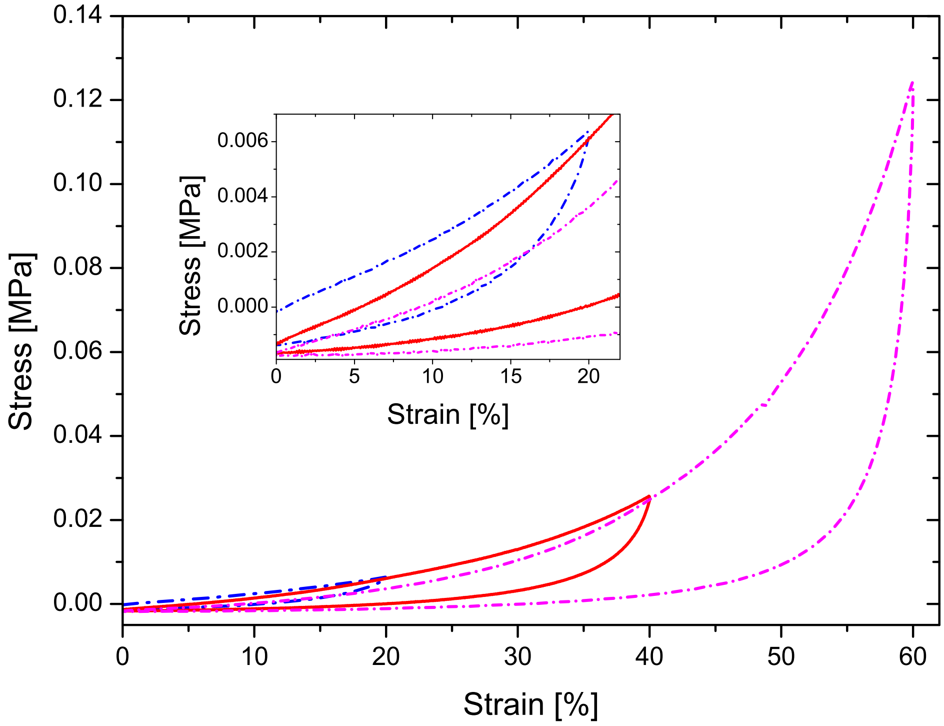

Figure S21. Stress-strain curves of TH under cyclic compression with stepwise increasing strain amplitude of 20%, 40% and 60% at -50°C

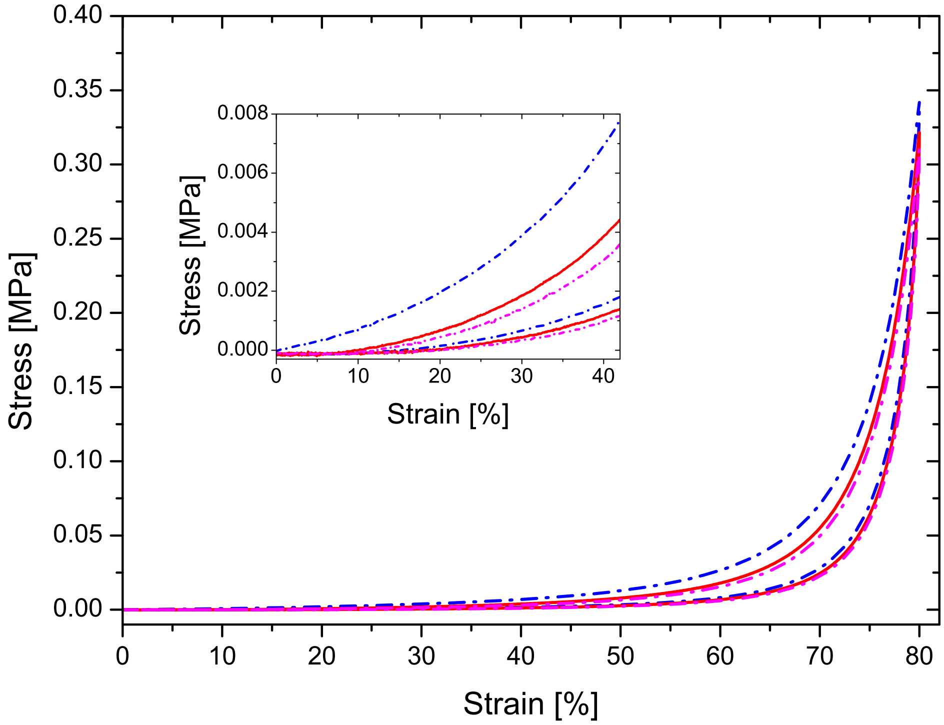

Figure S22. Stress-strain curves of TH from cyclic compression up to 80% strain, where every cycle was repeated three times, at 50°C

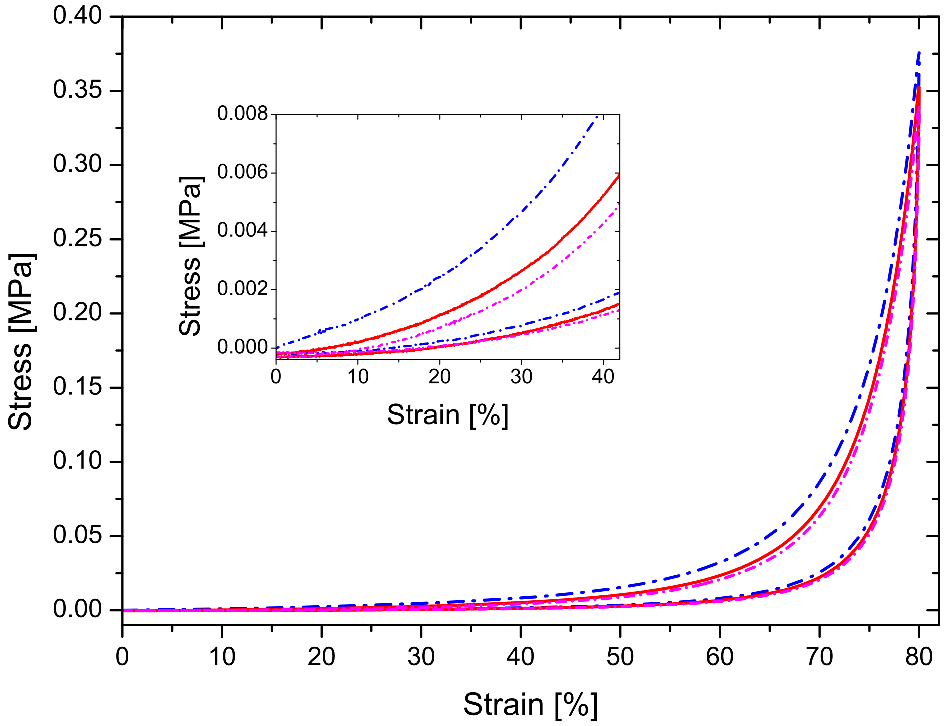

Figure S23. Stress-strain curves of TH from cyclic compression up to 80% strain, where every cycle was repeated three times, at 0°C

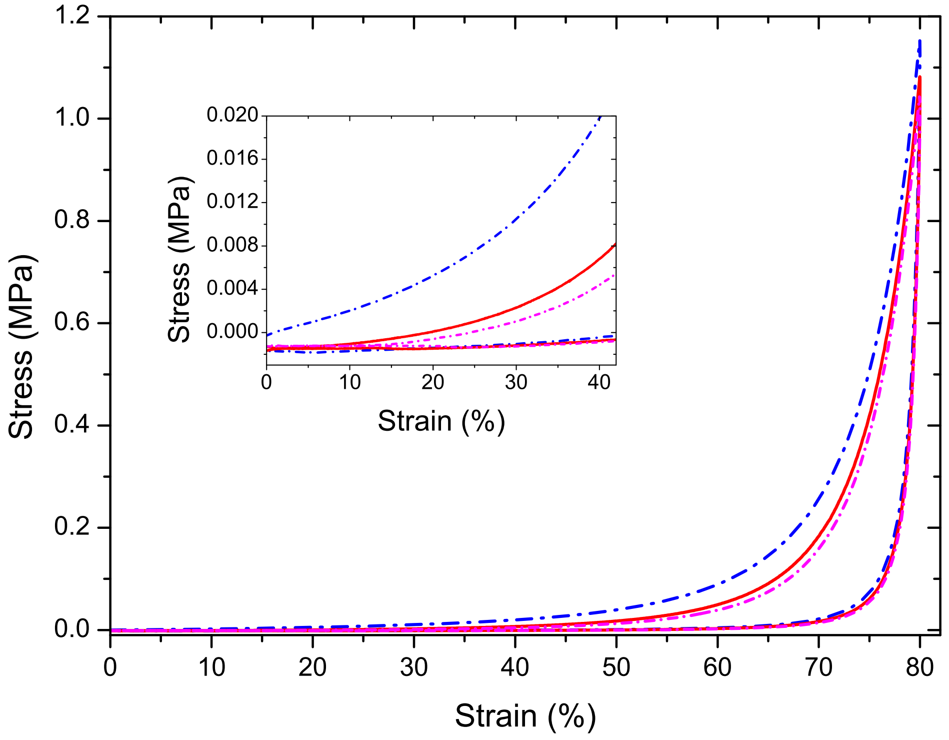

Figure S24. Stress-strain curves of TH from cyclic compression up to 80% strain, where every cycle was repeated three times, at -50°C
